# Settlement deservingness perceptions of climate change, economic, and political migrant groups across partisan lines

**DOI:** 10.3389/fsoc.2025.1510672

**Published:** 2025-01-30

**Authors:** Defne Aksit, Tijs Laenen

**Affiliations:** Centre for Sociological Research, Faculty of Social Sciences, KU Leuven, Leuven, Belgium

**Keywords:** settlement deservingness, deservingness theory, CARIN model, public opinion, international migration

## Abstract

International migration is a prevailing issue of our times. With opponents of multicultural societies becoming more vocal across Europe, it is pivotal to strengthen our knowledge of how migrants are popularly perceived in receiving countries. Prior research suggests that there is remarkable agreement within different countries as to which types of migrants are seen as deserving of settlement, cutting across deep-rooted partisan divides. Building on the CARIN deservingness theory, this article sheds new light on this so-called “hidden immigration consensus” by investigating Americans’ original perceptions of different migrant groups rather than following the standard practice of assessing how they react to a set of pre-defined migrant characteristics in a conjoint experiment. Based on a split-sample experiment, our results show that liberals and conservatives significantly differ in their perceptions of political, economic, and climate change migrants on four of the five CARIN criteria. Liberals differentiate between migrants on control, attitude, and identity criteria, whereas conservatives only distinguish on the control criterion. Liberals rate all migrant groups twice as deserving as conservatives. The implications for the settlement deservingness model and the hidden consensus hypothesis are discussed.

## Introduction

Migration is one of the great debates of our time. Across Europe, far-right parties utilize natives’ mounting fears around migration, especially that of being “replaced” to become formidable forces in the political landscape (Wodak, 2020); whereas in the U.S., the debate around migration only deepens the divide between Democrats and Republicans (Jost, 2006). The media images of desperate migrants on overstuffed boats trying to cross the Mediterranean and seemingly endless migrant caravans from South America piling up at the southern border of U.S. enforce the narrative that the world is in the midst of a genuine migration crisis.

Although there exist narratives against this alarmist view (De Haas, 2023), the issue still receives much attention, and immigration remains very much a salient and a thorny subject on the political agendas. In light of this, it is pivotal to comprehend how host populations perceive incoming migrants in terms of their deservingness to settle. While most countries have anti-discrimination laws in place to prevent this from happening, such popular perceptions often do determine how migrants are treated in civil society (for example on the housing or labor market) and in public policy (for example in immigration and welfare policies) (Böhmelt, 2021; Carlsson and Eriksson, 2017; Hubl and Pfeifer, 2013; Ubalde and Alarcón, 2020). Therefore, this article investigates how three of the main incoming migrant groups –i.e., climate, economic and political migrants– are popularly perceived by the American public: who is considered deserving to settle in the U.S., and why?

## Lacunae and objectives

Migrant deservingness is a subject that has been garnering increasing research attention. There exists evidence of a remarkable agreement across ideological and socio-structural cleavages as to which types of migrants should be allowed to settle in the host country both in the U.S. (Hainmueller and Hopkins, 2015) and in European (Bansak et al., 2016; Hedegaard and Larsen, 2022) contexts. This phenomenon is referred to as the so-called “hidden consensus.” Methodologically, these studies have mainly relied on conjoint experiments that investigate how members of the host population react to hypothetical descriptions of individual migrants that differ on a select number of characteristics such as their profession, whether they have already stayed in the U.S. in the past, language skills, and education. However, we argue that in reality the host population does not often receive such detailed information on individual migrants to base their deservingness perceptions on. Indeed, particularly in the U.S., exacerbated by the Trump presidential campaigns, the subsequent election victory of 2016, as well as the upcoming presidential election of 2024, the dominant rhetoric around migrants often emphasizes the size of the migrant group to imply migrants are a threat to the natives of the country [i.e., migrant “caravans invading” the U.S. (Fabregat et al., 2020)] and their reason for migrating is at the forefront (Pew Research Center, 2024a), rather than their individual characteristics. Our study aims to fill this gap in the literature by investigating how different types of migrants are *originally* perceived by the host population, without having received any pre-defined experimental stimuli about their individual characteristics.

Theoretically, we rely on the CARIN deservingness theory, which was originally developed to study welfare state attitudes (Oorschot, 2000) but has since been adapted to the migrant deservingness research (De Coninck et al., 2022, 2024). CARIN deservingness theory proposes that people utilize five criteria (control, attitude, reciprocity, identity, need) to form their deservingness judgments. The contribution of the CARIN framework is that it goes beyond ranking different types of migrants – economic, climate change, and political – on their overall perceived settlement deservingness, but also allows for a more in-depth investigation as to *which criteria exactly* affected this ranking. To this end, we investigate whether the hidden consensus that was established in the conjoint studies also hold true for the CARIN criteria. We assume exposure to widely diverging immigration frames in real-world partisan politics and media presumably makes right-wing conservatives and left-wing liberals evaluate various types of migrants differently on the five CARIN criteria (Jost, 2006).

Using a new split-sample experiment conducted among a sample of 762 Americans, we formulate our general research aim; to investigate how political liberals and conservatives perceive economic, climate change, and political migrants on the CARIN criteria.

We are interested in two concrete research questions:

*RQ1*: Do liberals and conservatives differentiate between different types of migrants on the CARIN criteria?

RQ1 thus focuses on the differences *between migrant groups* and investigates whether, e.g., liberals consider climate migrants to be less in control of their situation than economic migrants or whether conservatives believe political migrants are more grateful to settle in the country than climate migrants.

*RQ2*: Do liberals and conservatives differ from each other on CARIN criteria ratings for each migrant group?

RQ2 therefore focuses on the differences *between liberals and conservatives* and investigates whether, e.g., liberals consider economic migrants to be more in need than conservatives.

We sampled our participants from the U.S. in order to capitalize on the migration debate that is particularly politicized and is a driving force of polarization between the two ends of the political spectrum. In that sense, the U.S. is one of the least likely cases to find agreement between different partisan groups on the issue of migration, which is why prior research labels the apparent consensus between U.S. liberals and conservatives as a “hidden” consensus (Hainmueller and Hopkins, 2015).

## Theory and hypotheses

### The CARIN settlement deservingness theory

To answer our research questions, we turn to the CARIN deservingness theory. This theory was originally developed to assess people’s perceptions of which target groups in a society (e.g., the elderly, the poor, the unemployed, the infirm, immigrants) are deserving of welfare benefits and services. It is a descriptive rather than a normative theory (Meuleman et al., 2020) in that it describes how deservingness perceptions are formed instead of advocating for a certain deservingness morality. The underlying question it attempts to answer is “who should get what and why?” (Van Oorschot and Roosma, 2017, p. 3) with the goal of distinguishing between those who are considered worthy of welfare benefits and those who are not by employing the so-called *CARIN deservingness criteria*.

**Figure 1:**
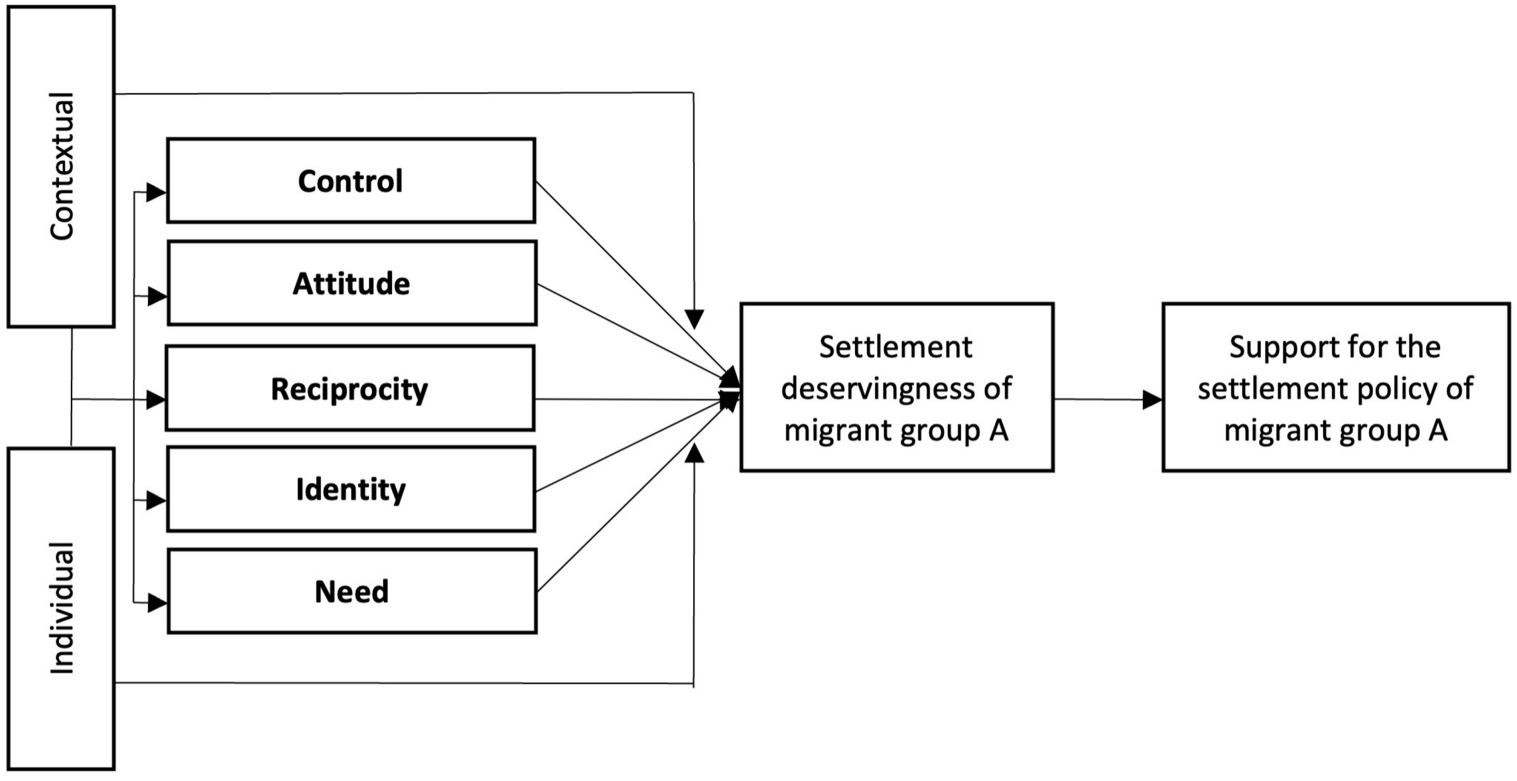
The CARIN settlement deservingness theory.

The five deservingness criteria are: Control, Attitude, Reciprocity, Identity and Need – which together form the CARIN criteria (Figure 1). The validity of the criteria for settlement deservingness has been confirmed in a cross-country survey (De Coninck et al., 2022), and re-contextualized accordingly (De Coninck and Matthijs, 2020). The control criterion draws from a person’s self-agency. If the target persons are perceived to have personal responsibility in finding themselves in an unfavorable position (e.g., being unwilling to work) as opposed to being a victim of an event outside their control (e.g., losing their job after a work injury), they are seen as less deserving (Laenen, 2020). A migrant would be considered more deserving if they are perceived as having migrated “involuntarily,” due to an external event they had no control over (like an armed conflict or a natural disaster), as opposed to voluntarily leaving their country, for example in search of better employment prospects or for family reunification.

The attitude criterion relates to the norm-complying behavior of the target groups (van Oorschot, 2006). Welfare recipients who are docile, grateful, pleasant, and overall likable are perceived to be more deserving than those who deviate from social norms and are “demanding” (Laenen, 2020). In the context of settlement deservingness, the attitude criterion can be re-imagined as the gratefulness a migrant group shows to be allowed to settle in the country. The criterion of reciprocity is concerned with people “earning” the help they receive (van Oorschot, 2006). Target groups are considered more deserving when (whether in the past, present or the future) they contribute to the society in some meaningful way such as by paying taxes. For migrants, reciprocity can be tied to their education level and labor market skills (De Coninck and Matthijs, 2020). As they are only arriving to the country, they have not contributed anything to the society in the past. Therefore, they are more likely to be judged on their potential *future* reciprocity (Laenen et al., 2019), where a high education and a large set of skills can make it easier for them to find employment and contribute to the wealth of the society in the future. The identity criterion captures target groups’ deservingness by the degree of similarity between the in-group and out-group (van Oorschot, 2006). The more the target group is perceived to belong to the in-group as opposed to the out-group, the more deserving they are perceived to be. Degree of similarity is conventionally conceptualized as race, nationality, and ethnicity (Laenen, 2020) but can be interpreted broader to include other characteristics such as age, gender, language and religion (Chen and Li, 2009). In the context of settlement, the degree to which a migrant group shows willingness to adapt to the host country’s norms and values can be seen as a reasonable measure of the identity criterion. The criterion of need, finally, refers to the perceived level of neediness, either financially or in terms of health (van Oorschot, 2006). The higher the level of neediness, the higher the perceived deservingness of the target group. For migrants groups, neediness can refer to financial and health issues, but can also include their need for protection.

Therefore, if a migrant group is perceived to be migrating for reasons outside their control (Control), grateful as opposed to demanding (Attitude), likely to contribute to U.S. society as opposed to being lazy (Reciprocity), are willing to adapt to U.S. norms and values as opposed to refuse cultural integration (Identity), having material needs as opposed to not being in need (Neediness), then according to the CARIN settlement deservingness model, this migrant group should be popularly perceived to be deserving of settlement in the U.S.

### Theoretical expectations

The goals of this paper are to investigate (RQ1) whether liberals and conservatives differentiate between migrants on CARIN criteria, and (RQ2) whether liberals and conservatives differ from each other on how they rate each migrant group on CARIN criteria. We investigate liberals and conservatives separately, because the CARIN model (Figure 1) assumes individual characteristics play a role in how CARIN criteria perceptions are formed. Political ideology is one such characteristic and should therefore affect CARIN criteria judgments. This reasoning stands in contrast to the hidden consensus hypothesis, which proposes an optimal migrant profile that transcends sociopolitical divides. While this comparison between the so-called optimal migrant profile and the CARIN criteria ratings are not exactly identical, as mentioned in the introduction, the underlying idea remains similar: liberals and conservatives agree on a set of migrant characteristics, despite their ideological differences. It is possible there exists some nuance, in that some migrant groups are rated similar on some CARIN criteria by both liberals and conservatives, and differently on others. Our research questions are thus mostly exploratory, as literature on migrant settlement deservingness generally focuses on the overall ranking of migrants in settlement deservingness where multiple migrant characteristics (that do not match up with the CARIN criteria) are manipulated at once (Hedegaard, 2022; Helbling, 2020). We do, however, formulate some theoretical expectations regarding two out of the five criteria.

#### Control

If a migrant group is perceived to be migrating for reasons outside their control or fleeing a hostile situation or environment they had no fault in creating, they are considered *involuntary* migrants. These migrants garner decidedly more sympathy and support from the general public, and are often considered “real refugees” whose claim to settlement in considered more legitimate because of their forced migration (Verkuyten et al., 2018). On the other hand, migrants who are perceived to be voluntarily migrating, who choose to settle in another country out of “their own free will” are often accused of playing the system to take advantage of better employment opportunities in other countries (Crawley and Skleparis, 2018) or “asylum shopping” to settle in the country with the most favorable social benefits (European Commission Migration and Home Affairs, n.d.). Mass media narratives then make a separation between the “real” refugees and opportunity hunting migrants, which has perpetuated the idea that real refugees can only be helped at the cost of withholding resources from the illegitimate migrants (Bilgen et al., 2023).

While political migrants and climate migrants fall into the involuntary migrant category, economic migrants are far more likely to be perceived as voluntary migrants (we purposefully did not use the word “refugee” to refer to the migrant group(s) that would qualify as such, so as to avoid the extra “advantage” the designation of a refugee would bring in increasing perceived deservingness). Therefore, we expect economic migrants to be considered the most in control of their situation compared to political and climate for both liberals and conservatives.

Although climate change and political migrants are both, by definition, fleeing a situation that they did not personally create and should therefore be considered low in control, we predict conservatives will rate climate migrants higher in control than liberals. We predict this will be due to the more widespread denial of man-made climate change among the U.S. conservatives compared to liberals (Pew Research Center, 2019). If conservatives do not believe in climate change, then they are also not likely to consider a group of migrants fleeing from it to be low in control.

#### Reciprocity

Reciprocity criterion is operationalized in this study as the future benefit a migrant group can provide to the country. As economic migrants relocate with the explicit intention of finding work as opposed to political and climate change migrants, it is possible that they would be perceived as the most reciprocal migrant type. However, a smaller portion of the American public also believe economic migrants are taking away jobs from native workers (Pew Research Center, 2020), which benefits only the migrants and harms the receiving country citizens. From this perspective, they can also be considered as the opposite of reciprocal and score the lowest on this criterion compared to climate change and political migrants. As for the effect of partisanship, there is evidence that political conservatives are more likely to believe immigrants are worsening the U.S. economy (Pew Research Center, 2024b). Therefore, we predict conservatives will rate economic migrants significantly lower on reciprocity than liberals.

Finally, we predict that all liberals will consider all migrant groups more deserving than conservatives. Prior research has shown that right-wing conservatives generally have more negative perceptions of different migrant groups than left-wing liberals, presumably because they have diverging predispositions and receive contrasting information cues from partisan politics and media. Indeed, the issue of immigration plays an increasingly polarizing role in U.S. politics (Brooks et al., 2016), with political ideology being a significant predictor of one’s stance on immigration (Jost, 2006). The divisive effect of immigration on liberals and conservatives is well documented (Ceobanu and Escandell, 2010; Gorodzeisky and Semyonov, 2009; Hainmueller and Hopkins, 2014; Sears and Henry, 2003). Accordingly, it is reasonable to expect that right-wing conservatives perceive migrants more negatively than liberals on overall settlement deservingness.

## Materials and methods

A quantitative split-sample survey experiment on Prolific was conducted to investigate the perceived settlement deservingness of climate change, political, and economic migrants. Prolific is a survey service that recruits participants according to the researcher’s specifications. It was chosen over other survey companies, such as MTurk, due to its fairer practices in participant compensation. Each CARIN criteria as well as the overall settlement deservingness perceptions were assessed with one item. Political ideology was measured as a binary variable (0 = liberal and 1 = conservative).

For each migrant category, the corresponding definition was given: *You will now be asked your opinion on migrants who come to US because*…

1. *they have been affected by the consequences of climate change in their country* (climate migrants)
2. *…they live in poverty in their own country* (economic migrants)
3. *…their lives are in danger in their own country* (political migrants).

The five CARIN criteria were measured using the following items: *To what extent do you think these migrants…*

1. *…left their country out of their own free will?* (Control)
2. *…would be grateful to settle in US?* (Attitude)
3. *…could make a meaningful contribution to the wealth of our society?* (Reciprocity)
4. *…want to adopt to US norms and values?* (Identity)
5. *…have serious material needs?* (Need)

The overall settlement deservingness perception (OSD) for each migrant group was measured on a scale of 0 to 100 with the item: *To what extent do you think these migrants should have a right to come to the US to live here?* The order in which the questions were presented was randomized for each group, except for OSD item, which was always presented after the items measuring the CARIN criteria.

Belief in climate change was measured with the item, *to what extent do you believe climate change is caused by human activity as opposed to natural processes?*

1. *Entirely by natural processes.*
2. *Mainly by natural processes.*
3. *About equally by natural processes and human activity.*
4. *Mainly by human activity.*
5. *Entirely by human activity.*
6. *I do not believe climate change is happening.*

G*Power was used to determine the necessary sample size. Based on a type 1 error of 5%, power of 85% and a small effect size of 0.05, 251 participants were needed for one condition. As there were no prior studies to base the effect size on, a small effect was chosen to be more conservative. As there were three conditions, 753 participants were required for this study. A total of 762 participants were recruited. Forty-nine participants were excluded from the analysis for completing the survey under 1 min (exclusion criterion as specified in the pre-registration). When participants first sign-up to be Prolific participants, they are screened by the Prolific screening service on their political ideology. As an extra precaution, since political leanings may change over time, the participants were asked if they identified more as a liberal, a conservative, or with another ideology prior to being directed to the survey. Those who answered “other” were screened out, as well as those whose answer did not match with the political ideology they had stated when they first signed up to be Prolific participants. A total of 532 participants identified as political liberals and 182 identified as political conservatives. The political skewness of the sample will be discussed later on. There were 233 participants in the political migrant condition (177 liberals, 56 conservatives), 241 participants in the economic migrant condition (72 conservatives, 169 liberals), and 239 participants in the climate migrant condition (186 liberals, 53 conservatives).

The survey was created on Qualtrics and administered on Prolific. The participation requirement was that respondents were living in the US. Participants were randomly assigned to one of three conditions and asked to fill out the survey. All participants read and agreed to an informed consent form prior to study begin. The survey took on average 3 min to complete. At the end of the survey, the participants were debriefed and paid for their time. This research was approved by the Ethical Review Board of Tilburg University (ERB code: TSB_RP931).

## Results

### Descriptive results

Table 1 shows the means and standard deviations of each migrant group on the five CARIN criteria and their OSD. Attitude criterion stood out from the rest, as it was almost always the criterion that the migrants scored the highest on. Both conservatives and liberals indicated they believe all migrant groups would be grateful to settle in the U.S., as indicated by scores above 71 across the board for conservatives and above 82 points for liberals. The migrant groups were also consistently rated high on the need criterion across political ideology, once again scoring above 70 points with the exception of climate migrants as rated by conservatives, where the climate change group scored a 64. Reciprocity criterion scores varied with political ideology. Liberals considered all migrant groups to be able to make meaningful contributions to the wealth of their country, whereas conservatives disagree with this point, as indicated by <50 points. The identity criterion in general produced the lowest ranking for all groups, suggesting there existed an overall sentiment that none of the migrant groups have a very strong wish to adopt to the U.S. norms and values. As for the control criterion, conservatives and liberals agree that political migrants are the ones least in control of their situation, but diverge almost 20 points on the case of climate migrants.

**Table 1:** Means and standard deviations of CARIN criteria and OSD.

	Climate migrants			Political migrants			Economic migrants		
	Liberals	Conservatives	Overall	Liberals	Conservatives	Overall	Liberals	Conservatives	Overall
	M (SD)	M (SD)	M (SD)	M (SD)	M (SD)	M (SD)	M (SD)	M (SD)	M (SD)
Control	48.87 (28.81)	67.66 (32.27)	53 (30.56)	42.5 (21.75)	56.98 (30.29)	46 (30.48)	62.17 (27.47)	76.56 (24.77)	66.4 (27.42)
Attitude	82.34 (18.02)	71.32 (27.13)	79.9 (20.84)	88.68 (13.89)	79.34 (21)	86.43 (16.33)	85.46 (15.81)	74.6 (26.57)	82.4 (20.11)
Reciprocity	77.04 (20.45)	43.15 (29.1)	69.5 (26.63)	77.38 (22.15)	47.07 (25.4)	70.1 (26.34)	76.23 (22.7)	45.86 (28.74)	67.2 (28.3)
Identity	57 (20.83)	37.19 (27.16)	52.6 (23.8)	58.13 (21.75)	41.29 (26.72)	54.1 (24.1)	63.23 (19.86)	43.38 (28.99)	57.27 (24.76)
Need	72.23 (21.1)	64.26 (29.91)	70.5 (23.5)	74.85 (22.6)	74.23 (22.04)	74.7 (22.4)	77.47 (22.76)	70.67 (27.08)	75.73 (23.84)
OSD	81.23 (22.64)	44.62 (32.11)	73.12 (29.26)	84.36 (21.03)	45 (31.36)	75 (29.2)	83.01 (22.93)	36.78 (32.88)	69.2 (33.72)
N	186	53	239	177	56	233	169	72	241

Overall, liberals consistently rated all migrant groups higher on each criterion than the conservatives, with the exception of the control criterion, where they consistently rated all groups to be lower in control (which is consistent with the pattern, as we have already theorized, a lower score on control criterion leads to higher perceptions of settlement deservingness). This trend was also reflected on the overall settlement deservingness perceptions, as liberals rated all migrants strongly deserving of settlement, regardless of their reason for flight, as indicated by the above 81 points each migrant group scored. Conservatives, on the other hand, rated each migrant group half as deserving as liberals, and in the case of economic migrants, even less than half. We now turn to ANOVA results to see if these differences are statistically significant.

#### Scores on CARIN criteria

RQ1 is concerned with how the migrant groups differ on the CARIN criteria. To answer RQ1, we conducted a one-way ANOVA with the migrant group as the grouping variable and the CARIN criteria as the dependent variables. The analyses were run for liberals and conservatives separately. Table 2 shows the results.

**Table 2:** One-way analysis of CARIN criteria for liberals and conservatives.

Ideology	CARIN criteria	F	*η* ^2^	*p*
*Liberals*		*F*(2, 531)		
	Control	21.1	0.074	<0.001*
	Attitude	7.085	0.026	<0.001*
	Reciprocity	0.127	0.000	0.881
	Identity	4.441	0.017	0.012*
	Need	2.492	0.009	0.084
*Conservatives*		*F*(2, 181)		
	Control	7.311	0.076	<0.001*
	Attitude	1.407	0.015	0.248
	Reciprocity	0.283	0.003	0.754
	Identity	0.77	0.009	0.465
	Need	1.972	0.022	0.142

^*^Mean difference is significant at the 0.05 level.

The results show that liberals differentiate between the migrant groups on the control, attitude and identity criterion, whereas conservatives only differentiate the migrant groups on the control criterion. *Post-hoc* tests were conducted for the CARIN criteria that were significant to investigate between exactly which groups these differentiations existed. Results are shown in Table 3.

**Table 3:** *Post-hoc* multiple comparison tests of CARIN criteria liberals and conservatives.

Ideology	CARIN criteria	Migrant type		Mean diff	*SE*	*p*	% 95 CI	
							LL	UL
*Liberals*								
	Control	Climate	Economic	−13.306	3.055	<0.001*	−20.49	−6.13
			Political	6.75	3.036	0.068	−0.39	13.89
		Economic	Political	19.703	3.089	<0.001*	12.44	26.96
	Attitude	Climate	Economic	−3.117	1.704	0.161	−7.12	0.89
			Political	−6.339	1.684	<0.001*	−10.3	−2.38
		Economic	Political	−3.222	1.725	0.149	−7.28	0.83
	Identity	Climate	Economic	−6.258	2.208	0.013*	1.05	11.46
			Political	−1.252	2.195	0.836	−3.91	6.41
		Economic	Political	−5.005	2.247	0.068	−10.77	0.28
*Conservatives*								
	Control	Climate	Economic	−8.901	5.203	0.204	−21.2	3.4
			Political	10.678	5.525	0.133	−2.38	23.74
		Economic	Political	19.58	5.122	<0.001*	7.48	31.68

^*^Mean difference is significant at the 0.05 level.

*Post-hoc* tests revealed liberals consider climate change and political migrants to be significantly less in control of their situation than economic migrants. Climate migrants were considered significantly less likely to be grateful to settle in the U.S. than political migrants, and they were also considered less likely to adopt to the U.S. norms and values. All migrant groups were considered equally likely to contribute to the society in the future and to have the same level of need.

Conservatives also considered economic migrants to be significantly more in control of their situation compared to political migrants, but this was the only differentiation they made between the migrant groups. According to the U.S. conservatives, climate change, economic and political migrant groups are all equally likely to feel grateful to be allowed to settle, contribute to the society to the same extent, are equally likely to adopt U.S. norms and values, and have a similar degree of need.

In sum, our theoretical expectations for the control criterion were partially confirmed, in that for liberals, we observed a ranking where economic migrants did indeed score the highest (i.e., the most in control) in the control criterion, followed by climate and political migrants. This ranking was partially observed in the conservative sample, as only the economic migrants were considered the least in control, with no statistical difference between the other groups.

As for the reciprocity criterion, contrary to our expectations, there were no differences observed between any of the migrant groups, neither for liberals nor for conservatives.

### Differences between liberals and conservatives

To answer RQ2, we conducted a one-way ANOVA for each migrant group with political ideology as the grouping variable and the CARIN criteria as the dependent variables. Table 4 shows the results. Across all migrant groups, liberals rated migrants to be less in control of their situation, more grateful to settle in the U.S., more likely to contribute to the wealth of the society and more likely to adopt U.S. norms and values than their conservative counterparts did. The level of need was the only criterion that liberals and conservatives partially agreed on: for economic migrants and political migrants, liberals and conservatives did not differ in the level of need they perceived. This was not the case for the climate change group, as liberals perceived this group of migrants to be in significantly greater need than conservatives.

**Table 4:** Differences between liberals and conservatives for each migrant group.

Migrant group	CARIN criteria	F	*η* ^2^	*p*
*Climate change*				
	Control	16.628	0.07	< 0.001*
	Attitude	12.065	0.05	< 0.001*
	Reciprocity	92.426	0.28	< 0.001*
	Identity	32.244	0.12	< 0.001*
	Need	4.807	0.02	0.029*
*Political*				
	Control	10.017	0.04	0.002*
	Attitude	14.728	0.6	< 0.001*
	Reciprocity	74.07	0.24	< 0.001*
	Identity	22.757	0.09	< 0.001*
	Need	0.032	0.000	0.858
*Economic*				
	Control	14.007	0.06	< 0.001*
	Attitude	14.169	0.06	< 0.001*
	Reciprocity	75.752	0.24	< 0.001*
	Identity	37.843	0.14	< 0.001*
	Need	3.034	0.01	0.083

*Mean difference is significant at the 0.05 level.

We had predicted that conservatives would rate climate migrants to be less in control of their situation as compared to liberals due to their lower belief in man-made climate change. While an independent samples *t*-test confirmed that liberals are significantly more likely to believe climate change is caused by human activity (*t*(58) = 5.497, *p* < 0.001, CI_95%_ = [0.658; 1.412]) than conservatives, belief in climate change did not predict conservatives’ perceptions of climate change migrants on the control criterion (*F*_(51, 1)_ = 3.606, *p* = 0.063, CI_95%_ = [−12.791; 0.355]).

Lastly, we tested the hypothesis which predicted that liberals would rate all migrant groups to be more deserving than conservatives. In line with our hypothesis, across all experimental conditions, liberals were almost twice as likely as conservatives to consider migrants deserving of settlement. Due to the unequal sample sizes and variance heterogeneity of groups, Welch’s *t*-test was run on all experimental conditions. The results showed that for the climate condition [*t*(64.866) = 54.832, *p* < 0.001], economic condition [*t*(104.721) = 129.096, *p* < 0.001], as well as for the political condition [*t*(73.741) = 76.491, *p* < 0.001], liberals rated all migrant groups significantly more deserving than conservatives; thus confirming our hypothesis.

## Discussion

This article investigated the perceived settlement deservingness of three broad migrant groups coming to the U.S and most other receiving countries: political, economic and climate change migrants. Building on the CARIN deservingness theory, we assessed Americans’ original perceptions of the level of control, attitude, reciprocity, identity and neediness of the different migrant groups and how these perceptions vary with political ideology. In doing so, we contribute to existing literature on migration attitudes in at least two important ways. First, by further substantiating CARIN deservingness theory as applied to migrant settlement deservingness, a framework that originates from the welfare attitudes literature (Oorschot, 2000) and has only recently been applied to the research on migration attitudes. Prior work on settlement deservingness (De Coninck et al., 2022, 2024; De Coninck and Matthijs, 2020), however, has mainly focused on the importance citizens attach to different criteria of deservingness rather than how they evaluate different migrant groups on these criteria. This distinction between deservingness *valuations* and deservingness *perceptions* is increasingly recognized in the research on welfare deservingness (Laenen, 2020), but has not found its way yet to the research on migrant settlement deservingness. Second, our analysis sheds new light on the so-called “hidden immigration consensus” identified in both American (Hainmueller and Hopkins, 2015) and European (Hedegaard and Larsen, 2022) research, which argues that there is broad agreement across partisan line as to which migrants should be allowed to settle in the receiving country. In the following, we discuss in turn our findings with regard to (1) deservingness theory and (2) the hidden immigration consensus.

Concerning the deservingness theory, conservatives and liberals differed in their ratings of all migrant groups across all the CARIN criteria, with the exception of the need criterion for political and economic migrants, with liberals perceiving all migrant groups on all criteria in a more positive light than conservatives. This divergence in opinion is reflected in the overall settlement deservingness perceptions, as liberals were, in all cases, twice as likely as conservatives to consider migrants deserving of settlement. This finding clearly supports the CARIN model proposition that individual characteristics impact how migrants are perceived on the CARIN criteria.

When it comes to differences in how migrant groups are perceived *in comparison to each other,* we found conservatives only make a distinction on the control criterion, where they consider economic migrants to be more in control of their situation. Liberals, on the other hand, make distinctions between migrant groups on three of the five CARIN criteria: they consider economic migrants to be more in control of their situation than climate and political migrants, consider climate migrants to be less grateful to settle in the U.S. and adopt the U.S. norms and values than political migrants. However, these differences in perceptions on the control, attitude and identity criteria, at the end, were not reflected in the overall settlement deservingness score in the sense that there was no significant difference in the degree to which liberals considered different migrant groups to be deserving of settlement. The same is also true for conservatives. This finding raises the question whether perceptions on how migrants score on different CARIN criteria actually affect the overall settlement perception at all. Based on the settlement theory, we expected that how migrants are perceived on different criteria would reflect on their settlement deservingness. We propose two possible explanations for our findings.

First, it is possible that a migrant group needs to *consistently* be perceived more negatively (or positively) than another group(s) on the CARIN criteria in order for these perceptions to affect the overall settlement deservingness. In our case, liberals distinguished more between migrant groups on the CARIN criteria than conservatives, and even then, on three out of the five criteria. Furthermore, these distinctions did not concern one migrant group consistently: economic migrants were perceived to be the highest in control, and climate change migrants were perceived to be lower in attitude and identity criteria than political migrants. It is possible that the reason these distinctions eventually did not show up in the migrant deservingness ratings is because a group needs to be *distinguished from other migrant groups on more criteria for it to take effect.* In other words, it could simply be that the different scores on CARIN criteria cancel each other out.

Second, the effect of partisanship may have been more powerful than the CARIN considerations. Liberals in general support a more open migration policy (Pew Research Center, 2022) than conservatives, which in the end may mean that they are less conditional on their settlement deservingness perceptions. So even though we did find differences in certain migrant groups on three CARIN criteria, at the end the importance liberals attach to how these migrant groups score on the CARIN criteria fades in the face of a generally accepting attitude toward migrants, regardless of what their personal circumstances may be. This is in line with findings from the welfare deservingness literature, where it has been established that left-wing liberals are known to discriminate less between different target groups, presumably because they have a greater ideological loyalty to the welfare state project (Laenen, 2020). Another finding from welfare literature also suggests that right-wing conservatives place greater weight on the issue of deservingness than their left-wing counterparts, making them more conditional in their solidarity with different target groups (e.g., Jeene et al., 2013; van Oorschot, 2006). Concretely, this means that right-wing conservatives make sharper distinctions between different groups as to who deserves help from the welfare state, which is a phenomenon we did not observe in our data, as conservatives rated all migrant groups to be equally (un)deserving and distinguished migrants on the CARIN criteria even less than liberals, namely only on control. This may be a mirror case of the liberals, where conservatives in general support a more restrictive migration policy, and therefore it makes little difference for them what the individual circumstances of a migrant group are; they would rather have no incoming migrants to begin with.

Our findings also have implications for the hidden consensus hypothesis, whose core finding is that across political divides, people converge on an “ideal” type of migrant by agreeing on certain characteristics. On the one hand, our study clearly shows that liberals and conservatives do not see eye to eye in how they perceive migrants on CARIN characteristics, in the sense that the former are twice as likely than the latter to consider the migrant groups as deserving of settlement. On the other hand, we cannot show that liberals and conservatives have different settlement rankings for different migrant groups, as for neither ideology there is a ranking to begin with. To be clear, this does *not* mean that prior research was wrong in suggesting the existence of such a consensus. When presented with experimentally manipulated profiles of individual migrants that have different pre-defined characteristics, liberals and conservatives do have similar relative preferences as to who should be allowed to settle in the country. Our argument here, however, is that it seems highly unlikely that citizens will receive such clear-cut immigration cues out in the real world, where the issue of immigration is usually framed in the context of partisan politics and media and different partisan groups are hence likely to diverge in their settlement deservingness perceptions.

### Limitations

A first limitation of our study refers to the specific context in which it was conducted. The U.S. is a country where the immigration debate—as have most other policy issues—is increasingly polarized along partisan lines. We leave it up to future research to explore to what extent our U.S. based findings are generalizable to other countries as well. While it seems safe to assume that such political polarization on the issue of immigration has taken place in most other receiving countries (e.g., Schmidt-Catran and Czymara, 2023) the U.S. could well be an exceptional outlier in this regard; making an immigration consensus less likely there. Furthermore, it seems plausible that the composition of the three migrant groups under consideration here –as well as the popular perception thereof– varies across countries. For example, while most Americans presumably think first and foremost of Mexicans when they consider economic migrants, many Europeans might have migrants from Sub-Saharan Africa in mind instead. In a similar vein, the (perceived) composition of the migrant categories can change over time, as happened with the European (but not the American) notion of a political refugee after the Russian invasion of Ukraine. Accordingly, it seems likely that citizens living in different countries (and times) will have different deservingness perceptions of the same migrant categories. Sticking to the example of the Ukrainian war, it could be that most Europeans now evaluate political migrants more positively on the criterion of identity, because this group is (perceived to be) composed of fellow Europeans that are closer in terms of ethnicity, language, religion, and other identity-based characteristics than, e.g., refugees from the Middle East (Sinclair et al., 2024). In the U.S., by contrast, this shift in the (perceived or real) composition of political migrants has presumably not occurred. Based on these considerations, we strongly encourage future (cross-national) research to investigate (using, e.g., an open-format manipulation check), who exactly respondents have in mind when answering questions about the deservingness of political, economic, climate migrants (see also Blinder, 2015). We further encourage future work to investigate the perceived settlement deservingness of other relevant migrant groups that were not considered in this article, including those who migrate because of family reunification or those who do so to for educational purposes.

Another avenue for future research is that migrants differ in other characteristics that were not measured in this study, notably their legal status and which generation of migrant they are. Different migrant generations often have access to different rights, as determined by their legal status and citizenship. Documentation and citizenship affect migrants’ ability to work, attend university, and their general participation in public life (Gurrola et al., 2016). Furthermore, legal status also affects how migrants are perceived by the general public. Murray and Marx (2013) demonstrated that participants consistently report higher perceived realistic threat, more prejudicial attitudes, and greater intergroup anxiety in response to questions about undocumented as opposed to documented migrants. Future studies should examine how migrants’ legal status interacts with their reason for flight (economic, political, or climate change) in informing public’s perceptions of migrant deservingness.

Finally, this study measured the “original” deservingness perceptions of migrants. How these perceptions are formed may be affected by several factors, including exposure to information on mass media. For example, as the volume of migration to U.S. rose over the last decades and the nationality of migrants have shifted to Third World countries, the public and policy attention on migration has focused on the costs the newcomers represent, rather than benefits associated with their arrival, particularly in the case of undocumented migrants (Bean et al., 1987). Such bias in mass media may affect how perceptions of migrant deservingness are formed and explain deservingness perception differences between migrants with different reasons for flight.

## Data Availability

The datasets presented in this study can be found in online repositories. The names of the repository/repositories and accession number(s) can be found at: https://osf.io/cbs9n/?view_only=d007776df5ba47d480132176b922a477.
